# Considerations in the Preclinical Assessment of the Safety of Antisense Oligonucleotides

**DOI:** 10.1089/nat.2022.0061

**Published:** 2023-02-01

**Authors:** Aurélie Goyenvalle, Cecilia Jimenez-Mallebrera, Willeke van Roon, Sabine Sewing, Arthur M. Krieg, Virginia Arechavala-Gomeza, Patrik Andersson

**Affiliations:** ^1^ Université Paris-Saclay, UVSQ, Inserm, END-ICAP, Versailles, France.; ^2^ Laboratorio de Investigación Aplicada en Enfermedades Neuromusculares, Unidad de Patología Neuromuscular, Servicio de Neuropediatría, Institut de Recerca Sant Joan de Déu, Esplugues de Llobregat, Spain.; ^3^ Centro de Investigaciones Biomédicas en Red de Enfermedades Raras (CIBERER), Madrid, Spain.; ^4^ Departamento de Genética, Microbiología y Estadística, Universitat de Barcelona, Barcelona, Spain.; ^5^ Department of Human Genetics, Leiden University Medical Center, Leiden, the Netherlands.; ^6^ Pharma Research and Early Development, Roche Innovation Center Basel, Basel, Switzerland.; ^7^ RNA Therapeutics Institute, University of Massachusetts, Worcester, Massachusetts, USA.; ^8^ Neuromuscular Disorders, Biocruces Bizkaia Health Research Institute, Barakaldo, Spain.; ^9^ Ikerbasque, Basque Foundation for Science, Bilbao, Spain.; ^10^ Safety Innovation, Safety Sciences, Clinical Pharmacology and Safety Sciences, R&D, AstraZeneca, Gothenburg, Sweden.

**Keywords:** antisense oligonucleotides, early preclinical safety assessment, toxicity, predictive assays

## Abstract

The nucleic acid therapeutics field has made tremendous progress in the past decades. Continuous advances in chemistry and design have led to many successful clinical applications, eliciting even more interest from researchers including both academic groups and drug development companies. Many preclinical studies in the field focus on improving the delivery of antisense oligonucleotide drugs (ONDs) and/or assessing their efficacy in target tissues, often neglecting the evaluation of toxicity, at least in early phases of development. A series of consensus recommendations regarding regulatory considerations and expectations have been generated by the Oligonucleotide Safety Working Group and the Japanese Research Working Group for the International Council for Harmonisation of Technical Requirements for Pharmaceuticals for Human Use S6 and Related Issues (WGS6) in several white papers. However, safety aspects should also be kept in sight in earlier phases while screening and designing OND to avoid subsequent failure in the development phase. Experts and members of the network “DARTER,” a COST Action funded by the Cooperation in Science and Technology of the EU, have utilized their collective experience working with OND, as well as their insights into OND-mediated toxicities, to generate a series of consensus recommendations to assess OND toxicity in early stages of preclinical research. In the past few years, several publications have described predictive assays, which can be used to assess OND-mediated toxicity *in vitro* or *ex vivo* to filter out potential toxic candidates before moving to *in vivo* phases of preclinical development, that is, animal toxicity studies. These assays also have the potential to provide translational insight since they allow a safety evaluation in human *in vitro* systems. Yet, small preliminary *in vivo* studies should also be considered to complement this early assessment. In this study, we summarize the state of the art and provide guidelines and recommendations on the different tests available for these early stage preclinical assessments.

## Introduction

Nucleic acid therapeutics (NATs) based on the use of antisense oligonucleotide drugs (ONDs) was, for many years, a niche for research in rare disorders, as traditional treatments were not available. However, considering that only a small fraction of the human genome has been successfully drugged to date [1], the approval of a dozen ONDs, including eight since 2018, has opened the doors to their application not only to a wider range of rare diseases but also to many common disorders. Because the mechanism of action of ONDs is related to the well-understood Watson–Crick–Franklin base-pairing rules, ONDs are straightforward to design, and this has increased the number of academic laboratories and small companies evaluating their potential to treat a myriad of disorders.

However, while many reference publications advise on how to design and evaluate the efficacy of these drugs, fewer address the evaluation of their safety and toxicological aspects. While many basic researchers may consider they are not likely to bring these molecules to a stage where formal safety assessment is required, there are several safety considerations that should be taken into account as early as possible during the discovery process. This would ensure correct interpretation of experiments and increase probability of success in the eventual future transition of a candidate molecule to clinical trials. Aspects such as the sequence, the chemistry of the backbone or the use of delivery systems to ensure efficient access to target tissues will influence the safety of these drugs and should be considered already at project initiation.

Broadly speaking, ONDs can be used to inhibit or restore the expression of some target genes by diverse mechanisms, including degrading messenger RNA (mRNA) transcripts causing gene silencing/knockdown or altering the splicing of pre-mRNAs [2]. For example, transcript degradation of mRNA can be achieved by antisense oligonucleotides (ASOs) recruiting RNase H1 cleaving DNA-RNA hybrids; by small interfering RNAs (siRNAs) that mediate target RNA degradation through RNA-induced silencing complex (RISC); or by splice-switching OND, which may be designed to skip regular exons in the pre-mRNA and thus create mRNA isoforms that encode nonfunctional proteins or trigger degradation of the mRNA by nonsense-mediated decay [3,4].

However, to restore functional protein expression in the treatment of disorders caused by splicing alterations, splice switching OND may be designed to promote exon inclusion, pseudo-exon exclusion, exon skipping, or splicing redirection in pre-mRNA [5]. More recently, ONDs have also been shown to upregulate gene expression by targeting nonproductive splicing events [6] or through the binding to regulatory elements, for example, upstream open reading frames [7,8].

As part of the endeavors of the network “DARTER,” a COST Action funded by the Cooperation in Science and Technology of the EU (www.antisenserna.eu), we aim to provide researchers with some guidelines on safety considerations during preclinical research, also called “discovery phase,” when designing these versatile drugs. A summary of the different assays that may be included from early on in the discovery phase is shown in Table 1 and expanded in the text of our article. In this article, we will focus on single-stranded ASOs, in particular phosphorothioate (PS)-ASOs because most of the published assays are described for that design, but most concepts also apply to other chemistries and mechanisms of action and we will provide examples for siRNAs when available.

**Table 1.** Summary of the Main Recommended Predictive Assays and the Associated References for Detailed Protocols

OND toxicity	Predictive assays	In vitro/in vivo	References for protocols
Off-target effects	*In silico* evaluation/*in vitro* validation	*In silico* and *in vitro*	Guidelines published by the OSWG in 2012 (Lindow *et al.* [16]; Michel *et al.* [146]; Yoshida *et al.* [147])
Immunostimulatory effects	• Quantification of cytokines/chemokines release in human PBMC or WBA by ELISA • Quantification of cytokines as well as CCL22 mRNA levels in BJAB cells by qRT-PCR	*In vitro*	Coch *et al.* [81]; Lankveld *et al.* [82]; Sewing *et al.* [104]; Anderson *et al.* [83]
Toxicities in high exposure organs	• Cytotoxicity by caspase assay Predictive assays for hepatotoxicity: • Quantification of LDH and ATP in primary hepatocytes • Caspase assay in transfected mouse 3T3 fibroblasts or human HepG2 cells • Evaluation of liver enzymes in mice/NHP Predictive assays for nephrotoxicity: • Quantification of EGF in human kidney tubule epithelial cells • Quantification of kidney injury biomarkers using chip-cultured HRPTEC • Quantification of urinary biomarkers (eg, β2-microglobulin and KIM-1) by ELISA	*In vitro* *In vitro* *In vitro* *In vivo* *In vitro* *In vitro* *In vivo*	Anderson *et al.* [83] Sewing *et al.* [104,105] Dieckmann *et al.* [94] Burel *et al.* [95] Moisan *et al.* [114] Nieskens *et al.* [115] Echevarría and Goyenvalle [117]
Thrombocytopenia	• Evaluation of platelet activation in human or NHP platelet-rich plasma or whole blood by flow cytometry (activation of CD62P and PAC-1)	*In vitro* and *in vivo*	Narayanan *et al.* [90]; Sewing *et al.* [71]; Slingsby *et al.* [85]
Inhibition of coagulation	• Quantification of PT and aPTT *in vitro* in human/mouse/NHP citrated plasma	*In vitro*	Echevarría *et al.* [122]; Relizani *et al.* [125]
Complement activation	• Quantification of split products of the APC (C3a, Bb, and C5a) *in vitro* in human/NHP/mouse serum	*In vitro*	Aupy *et al.* [123]; Henry *et al.* [129]; Sewing *et al.* [71]
CNS-specific toxicities	• Prediction of neurotoxicity from sequence features • Quantification of spontaneous calcium oscillations in primary cortical neuronal cultures	*In silico* *In vitro*	Hagedorn *et al.* [27] Hagedorn *et al.* [27]

APC, alternative pathway of the complement; aPTT, activated partial thromboplastin time; ATP, adenosine triphosphate; BJAB, EBV-negative Burkitt-like lymphoma cell line; CCL22, C-C Motif Chemokine Ligand 22; CNS, central nervous system; EGF, epidermal growth factor; ELISA, enzyme-linked immunosorbent assay; HRPTEC, human renal proximal tubule epithelial cells; KIM-1, kidney injury molecule-1; LDH, lactate dehydrogenase; mRNA, messenger RNA; NHP, non human primate; OND, oligonucleotide drug; OSWG, Oligonucleotide Safety Working Group; PBMC, peripheral blood mononuclear cell; PT, prothrombin time; qRT-PCR, quantitative reverse transcription-polymerase chain reaction; WBA, whole blood assay.

The target tissue and delivery route should also be carefully considered since high exposure organs will be different depending on local or systemic delivery. In this review, we will mostly discuss systemically delivered OND, which may accumulate in the liver or kidney, but we will also separately discuss the safety aspect of direct delivery to the central nervous system (CNS), which has gained increased interest over the past few years, especially since the clinical success of nusinersen.

## Overall Strategy and Considerations for OND Safety Testing

For OND projects aiming to reach the clinic, it is useful to understand the expectations of health authorities approving clinical trials. In 2020, the Japanese Pharmaceuticals and Medical Devices Agency issued a guideline for preclinical safety assessment of oligonucleotide therapeutics. In 2021 the U.S. Food and Drug Administration (FDA) issued a draft guidance, “Nonclinical Testing of Individualized Antisense Oligonucleotide Drug Products for Severely Debilitating or Life-Threatening Diseases,” providing a high-level overview of the key issues they will consider in the special situation of developing an OND to treat extremely rare mutations present on a single or very small number of patients (not for commercial development).

No specific formal guidelines for preclinical testing of ONDs to treat more common conditions have been issued to date by the International Council for Harmonisation of Technical Requirements for Pharmaceuticals for Human Use (ICH), FDA, or European Medical Agency (EMA), and so the testing principles normally follow the overall ICH M3(r2) guideline for small molecules. However, with great support from both sponsors and regulators, a series of consensus recommendations regarding regulatory considerations and expectations for OND have been generated by the Oligonucleotide Safety Working Group (OSWG) and other working groups in several white papers [9–20].

As briefly discussed in the Introduction, many academic researchers may not aim for clinical trials but “only” design and use ONDs to modulate targets to answer some specific biological questions. However, ONDs are not inert molecules and unless safety properties are understood and ideally selected for during potency screening of such research tools, scientists run the risk of incorrect interpretation of results due to undesired effects confounding the results. Although the requirements may be more rigorous for potential clinical candidates, the same principles and considerations apply for early safety assessment of tool compounds.

The main determinants of OND properties are chemistry, sequence, and design. For optimal performance, the choice of OND chemistry and design should be matched with intended use. Depending on ribose modification pattern, single-stranded ASOs with a PS backbone with a DNA gap can be used to reduce target transcripts via RNase H mechanism or without a DNA gap as steric blockers to modify splicing. With neutral backbone, for example, phosphorodiamidate morpholino oligomer (PMO) single-stranded ASOs can be used for splice modulation but not for RNase H mechanism.

RISC-dependent mechanisms such as target transcript degradation or microRNA mimics generally require a double-stranded design. In addition to mechanism, the chemistry and design also influence distribution and uptake. In general, single-stranded ASOs with PS backbone show productive uptake (leading to a pharmacodynamic effect) in several cell types on their own [21], whereas neutral backbone ASOs and double-stranded OND mostly need a delivery system or high doses for sufficient activity.

After having decided on the chemistry and design, the next step is screening for sequences with sufficient desired activity. Several guidelines have been published for OND sequence selection *in silico* [22–24]. Sequence motifs known to be associated with undesired safety effects should ideally be avoided already during the *in silico* design stage. Published examples of such motifs include cytosine guanine motifs (CpG) motifs activating Toll-like receptor (TLR)9 (immunostimulatory effects), polyG motifs, polypyrimidine, and other sequence motifs associated with toxicity in the liver [25,26] or neuronal toxicity flags [27]. Despite perfect homology, many sequences show poor activity so potency must be confirmed *in vitro* [28–30] using quantitative polymerase chain reaction for knockdown mechanisms followed by selection of the most potent sequences for further testing of for example, safety properties.

There are several points to consider when establishing assays and models for safety assessment of ONDs:

1. Is the assay intended for screening/filtering out undesired properties or for thorough characterization of for example, a clinical candidate?
2. Which reference ONDs should be used to validate the assay?
3. What should the study design look like?
4. Which are the best readouts?

Most established *in vitro* cellular models developed for safety screening of small molecules are either not relevant due to toxicity mechanism(s), not applicable for ONDs, or are not sensitive enough to pick up OND-induced toxicities. More complex *in vitro* models such as microphysiological systems (MPS) may be able to bridge this gap [31]. MPS are likely more reflecting the *in vivo* situation, but the cost and limited throughput will often prevent larger scale screening. At present, these models are likely best suited for characterization of a smaller number of really promising candidates.

Selection of *in vivo* models also needs careful consideration. Some safety concerns, such as proinflammatory manifestations, show relatively poor translation between species but may still be relevant to assess and select against to increase probability of success in regulatory toxicology studies. Moreover, it is not uncommon that ONDs showing good tolerability in naive wild-type animals trigger toxicity in disease models used for target validation and pharamokinetic/pharmacodynamic studies. To avoid confounding (toxicity) factors in such studies, safety characterization in the model of choice may be warranted before running larger pivotal studies.

The right reference compounds should be selected to validate and optimize the assay. These reference compounds should ideally be of the same chemistry and design as the OND aimed to be identified, for example, 3-10-3 locked nucleic acid (LNA)-DNA-LNA gapmer with full PS backbone modification or a GalNAc-conjugated fully 2′OMethyl modified 20mer PS backbone steric blocker. Smaller companies or academic research groups normally do not have a historic library of such reference compounds to use, but careful search of published articles can often identify some examples of safe and toxic reference compounds for a specific readout.

**FIG. 1.**
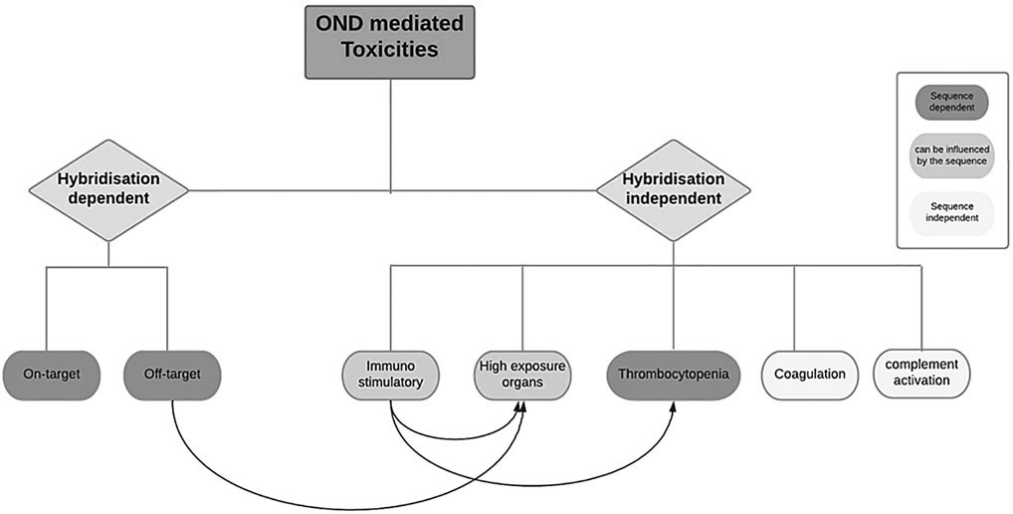
OND-associated toxicities: schematic representation of the most common OND-mediated toxicities. Some of these effects are strictly chemistry and design specific (sequence independent, *white boxes*), some are mostly class specific but can be influenced by the sequence (*light gray boxes*), and others are sequence dependent (*dark gray boxes*). The *arrows* represent the impact of a specific effect on another, for example, immunostimulatory effects play a role on thrombocytopenia and toxicities observed in high exposure organs. OND, oligonucleotide drug.

With a relatively slow onset to showing toxicity, OND safety *in vitro* and *in vivo* assays may require adapted study design, for example, longer duration than for other modalities. The safety considerations with ONDs can be categorized as: (1) hybridization and sequence dependent, (2) hybridization independent but sequence dependent, and (3) hybridization and sequence independent (“backbone effect”) (Fig. 1).

## Hybridization- and Sequence-Dependent Effects: On- and Off-Target Safety

### Potential on-target toxicities

Before a therapeutic candidate can move forward in the preclinical pipeline, its safety profile needs to be considered and optimized. This should start already during the selection of the intended target, by assessing the potential for so-called on-target toxicity or exaggerated pharmacology. On-target toxicity can result in too strong intended effect (eg, hypoglycemia induced by a diabetes treatment) or an adverse response to the drug in a tissue, which is not the intended target tissue. Although no clear examples of on-target safety issues have been published, ONDs can show a much longer effect duration compared with many other drug modalities: the washout period may be weeks or even months should adverse on-target effects be observed. For targets with clear on-target risk, strategies of oligo-based antidotes have been described [32,33], although those would require a dedicated safety assessment package before being applied in the clinic.

Until better tools are available, the assessment of on-target toxicity mostly relies on compiling available information on the target with regard to its biological function, tissue expression pattern, and from published information of other drugs for the same target to assess potential risks for patients. *In vitro* assessments ideally should be performed in biologically relevant cell lines or preferably, in primary human cells or MPS when possible, and *in vivo* testing is generally required. *In vivo* assessment requires a species cross-reactive OND or a species active surrogate molecule that is sufficiently potent and safe and should be of the same chemistry and design as the candidate OND. Detailed considerations for assessing on-target toxicities for OND can be found in the study by Kornbrust *et al.* [15].

### Potential off-target toxicities

However, hybridization-dependent off-target toxicities are those where ONDs act on transcripts other than the intended one via Watson–Crick–Franklin base pairing. These off-target effects may be severe, such as acute hepatotoxicity, and will be discussed further in the Toxicities in high exposure organs section. Several studies have described ways to assess hybridization-dependent off-target effects [34–38]. Some straightforward steps that should be followed when assessing off-target toxicities of gapmer ASOs using sequence alignment tools and sequencing data repositories (ie, transcriptomics) as summarized by the OSWG [16]:

1. Perform an *in silico* analysis (eg, Basic Local Alignment Search Tool search optimized for short sequences or other tools such as RNAhybrid [39]) of the entire pre-mRNA transcriptome (including both introns and exons) to search for potential complementary sequences (off-target candidates) using defined criteria. From the predictions, prioritize the sequences with no or as few predicted hybridization off-targets as possible. This should be the obvious approach for gapmer ASOs or siRNA where numerous sequence options exist but may be more challenging for steric blockers where choice of sequence is significantly restricted. For OND intended for clinical development, it may be a good idea to also assess and minimize potential off-targets for the species that will be used for regulatory toxicity studies. However, it should be kept in mind that sequence-based affinity predictors do not consider secondary structures nor RNA binding proteins that can influence the activity of ASO [40].
2. Reduce the list of potential off-target transcripts: for example, those expressed exclusively in organs with insufficient exposure, for example, CNS in the case of systemically administered PS backbone ASOs.
3. Perform *in vitro* concentration-response activity margin assessment in cells expressing both the primary target and the off-targets identified by *in silico* approaches using, for example, revese transcription-quantitative polymerase chain reaction or RNA-seq. The ASO exposure is ideally done by free (or “gymnotic”) uptake, but since all cell models may not support this, other delivery methods such as lipid transfection and electroporation can be considered, in which case the nonspecific impact of the delivery method should be taken into account.
4. The size of an acceptable *in vitro* margin depends on the potential consequence of meaningful modulation of the off-target at the intended dose levels. For off-targets lacking clear *in vitro* margins to primary target, follow the same principles as for on-target safety assessment, that is, assessing tissue expression profile, described biological role, pattern of developmental expression, effect of other compounds hitting the same target, and phenotype of genetically modified animals and human polymorphisms. Also, the potential of the off-target to trigger an adverse effect could be evaluated in relevant *in vitro* system (eg, MPS) and even *in vivo* using appropriate surrogate OND of the same chemistry and design targeting the animal version of the off-target.

In contrast to gapmer ASOs, ONDs that modulate pre-mRNA splicing by blocking access of splicing factors (general called steric-blocking oligonucleotides or SBO) may result in off-target events by causing unwanted splicing effects at near-complementary sites, or by causing changes in transcript levels (expression changes). A recent study analyzed the off-target splicing effects of 81 SBO and the off-target expression changes of a subset of 46 of those [41]. They found that off-target splicing effects are more predictable and predominantly hybridization dependent, whereas the off-target expression effects are less reproducible, although more frequent, and are probably driven by other mechanisms beyond hybridization such as transcriptional events and experimental variation.

Therefore, off-target expression effects of SBOs need to be investigated and validated *in vitro* in cells (and not only *in silico*) before being considered as bona fide off-target effects. *In silico* models to predict SBO off-target effects are often of limited use because they miss the majority of unwanted events. Therefore, conducting *in vitro* experiments such as RNA sequencing are preferable. Off-target hybridization-dependent splicing changes may be reduced by combining two SBOs at lower doses each, introducing mismatches and using shorter sequences [41]. The mode of delivery is also an important factor. Off-target mis-splicing events appear to be a greater problem *in vitro* when delivered to the cell by lipid transfection than *in vivo*, perhaps because *in vivo* there is a much lower amount of ASO available to reach the target [40].

When assessing potential hybridization-dependent on- and off-target toxicities for oligonucleotides, sequence differences may result in insufficient activity of the clinical candidate in animal test species. In such cases, surrogate ONDs designed for the test species (often rodent) may be developed and used in parallel to the human candidate to assess potential on-target safety in regulatory toxicity studies.

In addition, chemical modifications that protect against metabolizing nucleases result in slow tissue elimination and therefore in a longer duration of the effect of the ONDs. For example, the GalNAc-conjugated siRNA inclisiran shows maintained reduction of the target proprotein convertase subtilisin/kexin type 9 serine protease 6 months after a single subcutaneous administration. [42]. The potential mechanism for this observation has been described as a depot effect in lysosomes of the hepatocytes [43]. Although ASO gapmers show a shorter effect duration than siRNAs, several weeks are significantly longer than observed for most small-molecule therapeutics [44–47]. While this represents an advantage for patients, because less frequent administration is required, the sustained effect may require longer periods of time (weeks or months) for a potential adverse effect to resolve. Since long-term effects may be difficult to pick up *in vitro* in two-dimensional culture, this highlights the need for more complex system such as MPS, which can be kept in culture for several weeks [31].

Finally, the limited productive uptake of ONDs in certain cell types needs to be addressed when assessing hybridization-dependent on- and off-target safety risks. The limited productive uptake distribution often observed means that the real risk of any on- or off-target effect would be restricted to cell types with good productive uptake. For example, naked ASOs generally do not pass across the blood–brain barrier so the risk for on- and off-target toxicities in the CNS is rather negligible after systemic administration [21,45]. The tissue distribution of therapeutic oligonucleotides will vary depending on the administration routes and delivery systems such as conjugates or formulations, so understanding the productive uptake distribution for such new conditions is critical for proper risk assessment of potential on- and off-target toxicities.

## Hybridization Independent But Sequence-Dependent Toxicities

In contrast with on- and off-target effects, some toxicities are hybridization independent but still depend on the nucleic acid sequence. These include proinflammatory manifestations and effects in high exposure organs, such as the liver and kidney.

### Immunostimulatory effects

ONDs have long been recognized for their immune-stimulatory effects, which greatly depend on their design, chemistry, and nucleotide sequence [48,49]. ONDs can activate the innate immune system through binding to pattern recognition receptors (PRRs) such as the TLRs. While these immunostimulatory properties may be deliberately chosen for vaccine adjuvants or for cancer and autoimmune disease therapies [50–52], they are mostly undesired for other OND types.

The oligonucleotide sequence has been shown to be a key feature defining the immunomodulatory effects of ONDs. One of the best known example is the DNA CpG motif, which strongly activates TLR9 [53,54], particularly in the presence of optimal flanking sequences [55,56]. Examples of other proinflammatory sequences include RNA rich in guanine uracile motif or adenine uracil motifs, which trigger TLR7 and TLR8 responses [57,58] and U-rich sequences in siRNA [59–62]. The cytoplasmic PRRs such as retinoic acid-inducible gene I and protein kinase R are not activated by a specific nucleobase sequence but are instead triggered by structural features typical for viral RNA, such as the presence of uncapped 5′triphosphate in single- or double-stranded RNA, or blunt-ends in double-stranded RNA [63]. In contrast with proinflammatory sequences, some motifs have been associated with an immunosuppressant activity in particular for PS-containing ONDs [64–66].

To modify the immunostimulatory potential of a given sequence, chemical modifications have been introduced over the years. While it is commonly accepted that the PS modification has immunostimulatory effects [67,68], such effects have never been reported for OND with neutral backbones such as PMOs (at least when they are unconjugated) [69]. OND modifications frequently include 5′-methylation of cytosine residues to suppress the immune stimulatory effect of CpG DNA sequences [56,70]. Although ribose modifications such as 2′F, 2′methoxyethyl (MOE) and LNA were primarily introduced to increase affinity and further nuclease protection, their presence can also at least partly reduce the inflammatory stimuli [62,70,71].

Despite these efforts in design and chemical modifications, some of these modified ONDs still induce proinflammatory effects that can manifest in different ways in the clinic, including injection site reactions, flu-like symptoms, and thrombocytopenia [72–75]. Therefore, specific screening for immunostimulatory adverse effects is highly recommended in early preclinical stages to filter out potential proinflammatory candidates and prevent unexpected harmful effects in clinical development.

Rodents have been widely used for *in vivo* screening and they are considered particularly sensitive to immune stimulation, which does not always translate to humans. Treatment of mice with high doses of PS-ONDs has shown increased levels of circulating cytokines and chemokines [76,77] and resulted in a dose-dependent lymphoid hyperplasia with enlargement of spleen and lymph nodes as well as lymphohistiocytic cell infiltration often seen in multiple tissues [56,60,61,78,79]. In addition to *in vivo* assessment, *in vitro* tests have been designed to predict cytokine release using either isolated peripheral blood mononuclear cell or whole blood assay [71,80–82].

These offer the advantage of being species specific (human or non human primate [NHP]), which may be more relevant. Following incubation with various doses of ONDs, cytokines such as interleukin-6, interferon (IFN)-alpha, and tumor necrosis factor-alpha are generally measured in cell culture supernatants by enzyme-linked immunosorbent assay (ELISA) allowing fast, easy, and reliable predictions. An additional useful predictive assay was recently described using BJAB cells, an Epstein-Barr virus (EBV)-negative Burkitt-like lymphoma cell line, which is highly sensitive to stimulation by CpG and non-CpG oligonucleotides in a sequence and TLR9-dependent manner [83]. The induction of the cytokine CCl22 in OND-treated BJAB cells, measured by qRT-PCR, was shown to reliably predict the proinflammatory profile of gapmer ASOs.

### Thrombocytopenia

Thrombocytopenia, that is, decrease in platelet counts, has been occasionally reported in preclinical models (rodents and NHPs) and in three recent clinical trials following treatment with ONDs, in particular PS-ASO (volanesorsen, inotersen, and drisapersen) [84]. Two phenotypes of platelet count decrease have been distinguished: phenotype 1 characterized by a moderate but not clinically severe drop in platelet count and the rarer phenotype 2 of severe thrombocytopenia [85]. In contrast with PS-ASO, severe thrombocytopenia has not been reported for siRNA drugs, neither in preclinical studies nor in clinical trials, but encapsulation of siRNA in lipid nanoparticles has been shown to cause decrease in platelet counts in rats, presumably induced by the cationic lipid molecules themselves [86,87].

Following the observed results in humans with PS-ASO, retrospective analysis of NHP and human data performed by the company Ionis revealed that thrombocytopenia is sequence dependent and consistent across species (dose–response and time of onset). Approximately 40% of the 102 evaluated sequences induce a phenotype 1 platelet decline in NHP compared with 20% of 16 sequences observed in humans [88].

The underlying mechanisms of thrombocytopenia are still being investigated, and several immune- and non-immune-mediated hypotheses have been proposed. The presence of PS backbone has been shown to increase the risk of platelet activation [71,89]. Mechanistic investigations using non-CpG ASOs with therapeutically common modifications indicate no effect on the bone marrow or thrombopoiesis and have largely ruled out a platelet factor-4 (PF4) mechanism [71,90]. Instead, Narayanan *et al.* showed that thrombocytopenia is associated with increases in total immunoglobulin M (IgM), antiplatelet IgM, and/or anti-PF4 IgM and that monocyte activation contributes to increased platelet sequestration in the spleen and liver, leading to decreased platelets in peripheral blood [90].

More recently, the sequence-specific binding of OND (PS-ASO specifically) to platelet glycoprotein VI was shown to activate human platelets triggering formation of platelet–leukocyte aggregates [85]. These findings also highlight the possibility of a genetic susceptibility component given that donors with higher platelet glycoprotein VI levels had greater OND-induced platelet activation.

Overall, published studies and publically available data suggest that diverse risk factors are relevant for thrombocytopenic events observed in the clinic. Importantly, *in vitro* tools have been implemented to address some of the risk factors originating from the nature of the OND molecule early on at design and screening stage. Platelet activation can indeed be determined by measuring the activation of marker P-selectin (CD62P) and PAC-1 (activated GPIIb/IIIa) in platelet-rich plasma by flow cytometry [71]. Since *in vitro* platelet activation represents only a potential risk with no full validation of translation to thrombocytopenia in the clinic, these readouts should only serve as prioritization criteria for choosing the best OND from a pool of molecules with different behavior. This precaution is particularly meaningful in projects in which the route of administration and treatment duration entail a theoretical risk, that is, typically chronic systemic applications.

### Toxicities in high exposure organs

The highest concentrations of ONDs are generally found in the liver and kidney after systemic administration. The accumulation of ONDs *per se* in these organs is not necessarily associated with toxicities, which rather depend on the combination of OND sequence, chemistry, and designs. For ONDs of lower affinity chemistry (eg, 2′OMe, MOE), high doses leading to very high tissue concentrations are normally required to trigger kidney toxicity in preclinical species, but a different pattern is observed with ONDs of higher affinity chemistry [78], where there are examples of acute kidney injury in early clinical studies [91,92]. Importantly, gapmers using both moderate-affinity MOE and high-affinity LNA have caused unexpected renal toxicity in humans that had not been detected or predicted from the *in vitro* and *in vivo* screening assays run at the time [92,93], illustrating the importance of improved screening models for these effects, and the importance of monitoring relevant biomarkers in clinical studies.

As a first step to identify potential accumulation-driven cytotoxicity liabilities of ONDs, a caspase assay has been described using transient transfection or electroporation of ONDs in HepG2, 3T3, or Hepa1–6 cell lines and measuring caspase 3/7 activation [83,94]. This assay allows to deselect sequences with a high cytotoxicity liability early on before moving to more complex safety relevant *in vitro* models or *in vivo* studies.

#### Hepatotoxicity

Sequence-dependent toxicity of high-affinity ONDs has been observed in the liver relatively often during the discovery phase. In mice dosed only a single or a few times with some LNA or cEt gapmers, acute and subacute toxicities characterized by single cell necrosis, pronounced liver enzymes elevation, morbidity, and mortality have been reported [26,95,96]. Several studies have investigated these subacute toxicities and revealed different underlying mechanisms and identified sequence-specific motifs associated with hepatotoxicity [25,26].

A suggested mechanism involves PS backbone-dependent binding to key intracellular proteins in a sequence- and chemistry-dependent manner. Given the higher hydrophobic nature of high-affinity modifications, they also show higher affinity to a number of intracellular proteins compared with the same sequence with, for example, MOE chemistry [97–99]. Other molecular mechanisms have been proposed, including cell death as a cellular consequence to increased RNA degradation resulting from nonselective hybridization [71,94,95,100]. Indeed, hepatotoxicity was shown to be attenuated by RNase H1 knockdown [95].

In the case of siRNA, the hepatotoxicity observed in rodents has also been largely attributed to RNAi-mediated off-target effects [87], but not to the perturbation of RNAi pathways. This highlights the need to screen for hybridization-dependent off-target effects, as previously mentioned in the Potential off-target toxicities section.

Interestingly, it was recently shown that controlling PS stereochemistry in LNA gapmers or replacing PS in the DNA gap with methoxypropyl phosphonate or mesylphopsphoramidate can significantly improve their therapeutic index by improving safety without compromising activity [101–103], although this has not been tested in humans yet. In a similar detoxification effort, it was shown that the off-target effects observed with specific siRNA can be mitigated by modulating seed-pairing using a destabilizing chemical modification [36], analogous to placing an OMe in position 2 of the DNA part of gapmers [99].

In parallel to these design modifications reducing OND hepatotoxicity, predictive *in vitro* models have been established to filter out liver toxic candidates. The hepatotoxic potential of LNA gapmers can be evaluated in primary hepatocytes by measurement of extracellular lactate dehydrogenase and adenosine triphosphate levels following gymnotic delivery, as well as measurement of miR-122 expression in cell culture supernatants [104,105]. These studies showed that similar cytotoxicity readouts could be measured in mouse and human hepatocytes and correlated with the observed *in vivo* hepatotoxicity in mice, thus confirming the relevance of rodent hepatotoxicity to predict human hepatotoxicity.

#### Kidney toxicity

Following systemic administration, a large proportion of the administered dose of ONDs also end up accumulating in the kidneys, including the charge neutral backbones such as PMO [106,107], sometimes with kidney toxicity occurring. Renal lesions are generally restricted to the proximal tubules, in which the highest uptake is observed in contrast with kidney medulla [21,108]. Nonetheless, glomerulopathies were previously reported in mouse and monkey studies with the 2′OMe PS steric blocker drisapersen developed for the treatment of Duchenne muscular dystrophy [109], but these may have been linked to the chronic complement activation and inflammatory effects of the ASO. Rats develop a spontaneous lesion called chronic progressive nephropathy [110,111] that can be enhanced by kidney accumulation of PS backbone ASOs [78].

Renal toxicity was mostly regarded as an accumulation-related toxicity and primarily sequence unspecific until more acute tubular lesions were reported with high-affinity ONDs, such as LNA [112]. It has been suggested that these effects may be related to excessive accumulation of RNase H-dependent off-target transcripts and/or specific protein binding as described above for hepatotoxicity [113]. It is possible that the adverse sequence- and chemistry-dependent ASO:protein interactions proposed for liver toxicity also underlie the kidney toxicity observed for higher affinity modifications.

To screen out potential nephrotoxic candidates, several studies have reported useful predictive assays in the past few years. Moisan *et al.* identified the elevation of extracellular epidermal growth factor (EGF) as a robust and sensitive *in vitro* biomarker of LNA-induced cytotoxicity in human kidney proximal tubular epithelial cells [114]. More recently, another group explored the utility of *in vitro* systems to predict acute kidney injury and demonstrated cytotoxicity and induction in kidney injury biomarkers using chip-cultured human renal proximal tubule epithelial cells [115].

Besides these *in vitro* predictive assays, several specific early biomarkers of toxicity can be evaluated *in vivo* in mice (treated with high doses of ASO) to predict toxicity in preclinical development and exclude nephrotoxic candidates before moving forward to larger safety studies [109,116]. Urinary biomarkers of kidney toxicity can be quantified in urine collected from treated mice either shortly after ONDs injection to evaluate the potential acute kidney toxicity or after several weeks of repeated treatment to assess the potential long-term renal toxicity induced by the accumulation of ONDs in the kidneys.

These biomarkers include general ones such as total protein, albumin, or creatinine as well as specific kidney injury biomarkers such as β2-microglobulin, renin, kidney injury molecule-1, IFN-gamma-induced protein 10, Cystatin C, EGF, Lipocalin-2-NGAL, clusterin, and osteopontin as described in the studies by Echevarría and Goyenvalle [117] and Sandelius *et al*. [118]. Finally, more general biomarkers of renal toxicity can also be measured in the serum or plasma of mice treated with high doses of ONDs, such as urea, albumin, creatinine, and total protein.

Importantly, the development of predictive *in vitro* models of kidney toxicity and the improvement in OND design have allowed identification of potent ONDs with significant reduction in toxicity as illustrated by the development of GalNAc conjugates, which conferred a more favorable safety profile at the cellular level [119]. However, one should keep in mind that translation of such findings may be limited due to breakdown of conjugates *in vivo* resulting in the unmodified sequence of the OND with all its potential renal safety liabilities.

## Sequence- and Hybridization-Independent Effects

Besides these hybridization-independent toxicities that are impacted by the sequence, other toxicities are independent of both hybridization and sequence. These toxicities are generally driven by the plasma concentration reaching above a threshold level and include prolongation of coagulation time and activation of the alternative complement system. These findings can occasionally be observed in clinical studies but can be reduced by adapting dosing regimen and are in most cases of low magnitude with limited impact of clinical safety.

### Inhibition of coagulation

Prolongation of coagulation time often observed with PS-ASO results from the PS backbone interacting with the Tenase complex in the coagulation cascade [120,121]. It is considered a class effect, modulated by interactions of the OND with plasma proteins in a sequence-independent way. At low plasma concentrations, the PS modification selectively prolongs the partial thromboplastin time. However, at high plasma concentrations, both the intrinsic and extrinsic pathways are affected, suggesting additional inhibitory effects [120]. Prolongation of clotting times can be screened relatively easily both *in vivo* and *in vitro* in mouse, NHP, and human serum [88,122]. Following incubation of the OND in citrated serum, both the prothrombin time and the activated partial thromboplastin time can be measured as described in the studies by Echevarría *et al*. [122], Aupy *et al*. [123], Henry *et al*. [124], and Relizani *et al*. [125].

### Complement activation

Systemic administration of PS containing ONDs has been reported to elicit the complement alternative pathway due to plasma protein binding [126,127]. Although this hybridization-independent effect is mainly related to the PS backbone of single-stranded ASOs, unexpected complement activation has been observed with some specific sequences in the case of tricyclo-DNA for example [123]. Single-stranded PS backbone ASOs interact directly with plasma factor H (a negative regulator of the complement cascade). This interaction has been shown to reduce the free levels of inhibitor, leading to an uncontrolled amplification of the cascade and a release of split products such as Bb and anaphylatoxins C3a and C5a [126]. Repeated activation of the complement induced by chronic dosing of PS-ONDs can result in C3 depletion, eventually leading to altered complement function, secondary inflammation, and vasculitis [112,113,128].

While humans appear less sensitive to complement activation than NHP [14], it is recommended to routinely assess complement activation in preclinical safety studies of new OND candidates in NHPs to minimize complement-driven toxicities in NHP of longer duration. Early preclinical safety assessment can also be performed in mice to screen out particular toxic candidates as it was previously shown with tricyclo-DNA sequences [123]. More importantly, protocols to assess complement activation *in vitro* have been described in several studies, allowing a relevant assessment directly in human serum. Increasing concentrations of the candidate OND are generally incubated in serum, plasma, or whole blood from mice, NHP, or human, and the different split products of the alternative complement pathway (Bb, C3a, and C5a) are then measured by ELISA [71,123,129].

Since these effects on complement and coagulation are driven by reaching above a threshold level in plasma, they are generally transient in nature and disappear when the OND is cleared from plasma by tissue uptake, within hours from systemic administration [44–47]. Moreover, the dosing regimens are often adapted in the clinic (favoring slow intravenous infusion to bolus administration, for example) leading to reduced plasma concentrations and therefore rarely exceeding activation thresholds [128,130,131]. However, these effects should be carefully considered in toxicity studies in which much higher doses are administered, in particular in Cynomolgus monkeys, which tend to present higher sensitivity [112].

## Toxicities Associated with CNS Local Delivery

ASOs are generally charged and have a molecular weight of ∼5,000 to 10,000 Da: too large to cross the blood–brain barrier by simple diffusion and reach an effective concentration in the brain or spinal cord. Currently, the most frequently used CNS administration route in humans is intrathecal (IT) administration, and in rodent models intracerebroventricular delivery (ICV). This results in an immediate high and long-lasting ASO concentration in the cerebral spinal fluid and brain and significant pharmacodynamic effects up to 6 months after the last dosing [132–134]. Although immediately after both IT and ICV administration, there is a peak in systemic exposure, this peak lowers rapidly after dosing.

Moreover, the overall doses administered are lower compared with systemic delivery, together making the risks for peripheral toxicity lower for CNS applications [45]. However, some acute neurotoxic effects have been reported after ASO administration in rodent brain, and at least some of the potential on- and off-target toxicities associated with systemic delivery also appear to occur in the CNS [135]. This means that most of the previously mentioned assays are also useful to predict the safety profile of ONDs aimed for CNS delivery (eg, assay predicting proinflammatory profile); however, some specific toxicities have been observed and reported in rodents following CNS direct delivery.

ICV administration of ASOs has been shown to induce an immune response in rodent brain [136,137] that could persist up to 2 months after the last administration. This suggests a prolonged immune response to the treatment, and as described in the previous paragraph, these immune effects are dependent on sequence and chemical modifications of the OND.

The most overt neurotoxicity in preclinical rodent studies is the occurrence of slight tremors and seizure-like activities immediately after OND administration. In this context, sequence-specific toxicity was studied following CNS delivery by Hagedorn *et al.* where acute tolerability behavior was assessed 1 h after ICV bolus injection in the mouse brain of 148 different LNA-modified ASOs with full PS backbone. They found that in particular the number and position of guanine nucleotides in the 3′-end of the ASO increased toxicity, while there was a decrease when adenine nucleotides were substituted [27]. Recent studies demonstrated that reducing the PS content of ONDs leads to increased tolerability in the CNS [138,139].

Despite these advances in safety assessment, it remains difficult to predict human toxicity from animal models. This has again be highlighted for instance by the recently stopped large multinational phase 3 study—GENERATION HD1 trial (NCT03761849) investigating a gapmer ASO lowering both wild-type and mutant huntingtin protein [140]. In March 2021, dosing in this trial was stopped because the high-dose patient group performed worse on clinical rating scales and had higher frequencies of serious adverse events. Ongoing studies are aiming to better predict neurotoxicity with human-based cell models, in line with the European Union directives on animal use in science to advance the development of alternative model systems to replace animal studies [141].

For an OND-based approach that targets (pre) mRNA, the use of a human model is essential since there are considerable differences in expressed RNA between mouse and human [142] as well as in neuronal versus non-neuronal cells. In a study using human fibroblasts and nusinersen, the only FDA- and EMA-approved OND for a CNS disorder, the off-target effects of the splice modulating therapeutic OND nusinersen caused widespread alterations in gene expression (including innate immunity) and aberrant splicing [143]. However, the interpretation of RNA sequencing studies after OND delivery in cell and animal models is still challenging, distinguishing between off- and on-target effects as well as acute and long-term effects.

Primary cortical neuronal cultures were used to evaluate more than 1,600 LNA-modified PS ASO, using spontaneous calcium oscillations as a readout. This *in vitro* assay was found to accurately predict acute neurotoxicity found in mice [27]. Using human induced pluripotent stem cell-derived neuronal models for the *in vitro* assessment of seizure liability is promising for ONDs [144,145]. More studies where neurotoxicity is assessed both in animals and in human-based cell models will be needed to better predict OND neurotoxicity.

## Conclusions

**FIG. 2.**
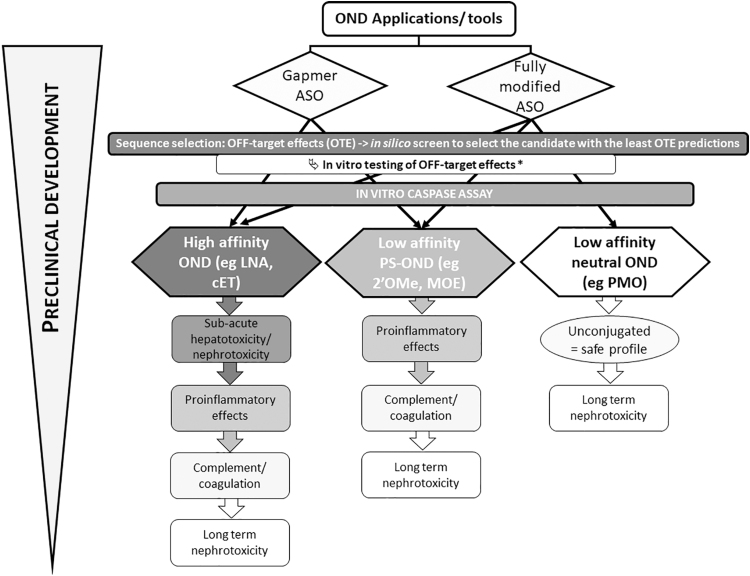
Simplified recommendation path to assess OND-associated toxicities depending on the design and chemistry: for all types of ONDs, it is highly recommended to perform *in silico* analysis during sequence selection to select the candidates with the least predicted OTE, followed by *in vitro* testing of the predictive OTE at a suitable time in the screening cascade. *Depending on the resources available, the *in vitro* screening may be run on a smaller set of candidates, for example, after proinflammatory assessment in the cascade. The overall cytotoxicity of OND can then be assessed *in vitro* using caspase assays, which offer good throughput to select safe compounds. Depending on the application and chemistry selected, the priority for predictive assay is then different since high-affinity ONDs present higher probabilities of inducing subacute hepatotoxicity or nephrotoxicity than low-affinity PS-ONDs or even ASO such as PMO, which have never been reported to induce such toxicities (when unconjugated). ASO, antisense oligonucleotide; cET, constrained ethyl; LNA, locked nucleic acid; MOE, methoxyethyl; OTE, off-target effects; PMO, phosphorodiamidate morpholino oligomer; PS, phosphorothioate.

OND-associated toxicities depend on sequence, chemistry, design, dose, duration, and the delivery route. We mostly focused here on systemic administration and direct delivery to the CNS and summarized the main type of toxicities than can be observed in preclinical research. More importantly, we have provided a list of predictive assays available to filter out potential toxic candidates during this preclinical research (Table 1). We also aimed to recapitulate the most commonly encountered types of toxicity depending on the mode of action (mostly described for RNase H-dependent ASO gapmer or steric blocking ASO) and the chemistry (high, medium/low affinity) to help scientists prioritize the type of assay for their preclinical assessment (Fig. 2).

High-affinity chemistries on PS backbone are indeed more likely to trigger subacute hepatotoxicity or nephrotoxicity, compared with chemistries with more moderate affinity (such as MOE and Me modifications). In contrast, charge neutral PMO has never been reported to induce such effects, but this may not hold true when PMO is conjugated or formulated to enhance their uptake. The last few years have witnessed tremendous progress in delivery systems aiming at improving the distribution of ONDs to target tissues.

These include development of lipidic ligands, peptide and antibody conjugates, or lipid-based nanoparticles. In this context, early preclinical assessment of safety aspects is crucial to ensure their future transition to the clinic. Far too often, preclinical studies focus on assessing only the improved delivery and efficacy, neglecting the importance of thorough early toxicity evaluation. With these guidelines, we hope to ultimately improve the success rate in the development phase and avoid safety signals in regulatory toxicity studies and ultimately in clinical studies that could have been identified earlier on.

So far, most ONDs have been developed to treat rare genetic disorders without existing therapeutic options. This has allowed some degree of acceptability of safety findings in terms of risk–benefit, but this assessment will likely be different in the coming years given the growing interest for ONDs and their development for much more common diseases for which therapeutic alternatives are available.

However, in parallel, our increasing understanding of the mechanisms underlying these various toxicities and the development of more complex *in vitro* tissue models will likely lead to more predictive and more sensitive test systems in the coming years. This will further improve the process to identify potent and safe ONDs and facilitate NAT drugs to reach their full potential.
